# The relationship between social media use and loneliness across the lifespan in the United States: Population-based study using Health Information National Trends Survey data

**DOI:** 10.1016/j.apro.2025.100196

**Published:** 2025-09-05

**Authors:** Christian E. Vazquez, Derek Falk, Dana P. Urbanski, Katherine Kwong, Diana Abudu-Birresborn, Juanita-Dawne Bacsu, Moka Yoo-Jeong, Hye Won Chai, Wonkyung Jung, Matthew Lee Smith

**Affiliations:** a School of Social Work, The University of Texas at Arlington, 501 W. Mitchell Street, Arlington, TX 76019, USA; b Department of Speech, Language and Hearing Sciences, College of Arts and Sciences, Indiana University Bloomington, 2631 E Discovery Pkwy, Bloomington, IN 47408 USA; c Department of Human Development, Connecticut College, 270 Mohegan Ave Pkwy, New London, CT 06320, USA; d Department of Nursing, Brock University, Niagara Region, 1812 Sir Isaac Brock Way, St., Catharines, ON L2S 3A1, Canada; e School of Nursing, Thompson Rivers University, 805 TRU Way, Kamloops, BC V2C 0C8, Canada; f School of Nursing, Bouvé College of Health Sciences, Northeastern University, 336 Huntington Ave 106J, Boston, MA 02115, USA; g Institute for Engaged Aging, Department of Psychology, Clemson University, 298 Memorial Dr, Seneca, SC 29672, USA; h Connell School of Nursing, Boston College, 140 Commonwealth Ave, Maloney Hall 346A, Chestnut Hill, MA 02467, USA; i Department of Health Promotion, Center for Community Health and Aging, School of Public Health, Texas A&M University, 212 Adriance Lab Road, College Station, TX 77843, USA

**Keywords:** Loneliness, Age cohorts, Social media, HINTS, PROMIS social isolation

## Abstract

**Objectives:**

Loneliness can affect all age groups and is continuing to grow as a major public health issue. One approach that has been proposed to address loneliness involves digital inclusivity because the ubiquity of technology and social media provides new avenues for activities to satisfy individuals’ needs for social connection. It is unclear which age groups could benefit more from using social media related to loneliness. The current study advances the limited research on the relationship between social media use and loneliness looking across multiple age groups with nationally representative data.

**Methods:**

Data were analyzed from the 2022 Health Information National Trends Survey (HINTS; n = 4774). This study used multiple linear regression stratified by four age cohorts (Millennials, Generation X, Baby Boomers, Silent Generation) to examine the association of social media use with PROMIS Social Isolation scores and to assess differences across age cohorts. Covariates included age, gender, race and ethnicity, education, income, living with other adults, marital status, having a friend to talk to about health, self-rated health, PROMIS Meaning and Purpose, and the PHQ-4.

**Results:**

For Baby Boomers, compared to never using social media, using social media daily (*b*=1.53, *p* = .008) was associated with a higher loneliness score. For the Silent Generation, compared to never using social media, using social media daily (*b*=2.43, *p* = .04) was associated with a higher loneliness score. There was not a significant relationship between social media and loneliness for Millennials and Generation X.

**Conclusions:**

Social media use may be a risk factor for loneliness particularly for those who are from the Baby Boomer and Silent Generation and use social media daily. Social media interventions aimed at decreasing loneliness among older adults should consider the directional of this relationship, which needs to be confirmed.

## Introduction

Social isolation and loneliness have gained global attention as public health issues due to their increasing impact on the health and well-being of individuals [1]. This is an issue that affects all age groups as current domestic reports estimate that about 25 % of older adults and about 40 % of young adults experience social isolation or loneliness [2]. There are mixed findings about the relationship between age and social isolation or loneliness. Some studies suggest a non-linear relationship, (e.g., lower rates around age 30 and age 60; higher in youngest and oldest adults); [3,4], while others suggest a more positive or linear direction [5,6]. In response to the growing concern and impact of social isolation and loneliness, there have been policy initiatives developed by the World Health Organization (WHO) to raise awareness and recognize social isolation and loneliness as a public health priority [7]. One of the proposed approaches to addressing this issue involves digital inclusivity [8].

While social isolation and loneliness are closely related, they are distinct constructs that can co-occur or exist separately [9]. Loneliness is a subjective feeling of isolation while social isolation is an objective state of isolation indicated by limited social contact or network size [10,11]. Both have implications for individuals’ social, physical, and mental health [12,13]. An additional challenge with the distinction between the two is the variation in measures used to evaluate social isolation and loneliness, which are sometimes discussed interchangeably [14]. The current study used the Patient-Reported Outcomes Measurement Information System^®^ (PROMIS, https://www.promishealth.org/57461-2/) measure of social isolation, which measures perceived social isolation (a.k.a., loneliness). In the current study, though the measure is named PROMIS social isolation, we used the term loneliness to refer to this construct, as individuals’ responses on this PROMIS measure reflect subjective feelings of isolation.

Technology and social media provide new avenues for activities to satisfy individuals’ basic needs and desires for social connection [15], prompting calls for digital inclusivity to address loneliness [8]. Research shows that using social media to communicate with friends and family can enhance a sense of belonging [16,17]. However, limitations in digital literacy and challenges with overstimulation may lead to poor or limited meaningful engagement, heightening and exacerbating loneliness [13]. Evidence suggests that among older adults, social media use is associated with reduced social isolation and loneliness [13,18–20]—whereas in younger adults, social media use has been linked to higher levels of social isolation and loneliness [21–23]. These age differences may be partly explained by other factors such as: access and digital literacy, social needs, and cognition [24–27].

The literature has used non-representative samples or only focus on specific age groups [28,29]. The current study fills three gaps in the literature about the relationship between loneliness and: a) social media use nationally, b) age nationally, and c) the intersection of age and social media use nationally. Filling these gaps can inform what is known about the relationship between social media use and loneliness [30–32]. Given the mixed findings on the relationship between social media use and loneliness when comparing across age groups and the lack of nationally representative studies that examine this relationship across multiple age cohorts, the current study was conducted to address the following research questions:

1. Is social media use associated with loneliness?
2. Does the relationship between social media use and loneliness vary across age cohorts?

## Methods

### Data sources

Data were analyzed from the 2022 Health Information National Trends Survey (HINTS). HINTS is a nationally representative survey of the noninstitutionalized, adult population (aged ≥18 years) in the United States [33]. HINTS is an assessment of the American public’s access to and use of information about the cancer care continuum, which spans from prevention and early detection to diagnosis, treatment, and survivorship. In addition to this standard HINTS content, the survey also includes questions related to general eHealth information-seeking behaviors not specific to cancer.

In 2022, questions from the National Institutes of Health (NIH) PROMIS were added to the survey. Data collection for the 2022 survey cycle began on March 7, 2022 and concluded on November 8, 2022 (N = 6252), with a response rate of 28.1 %. Respondents were sampled using a complex sampling design to achieve a nationally representative sample. Data were gathered using web-based surveys and surveys sent by mail. Surveys were available in English and Spanish. Detailed information about the design, procedures, measures, and weighting of HINTS can be found elsewhere [33]. Institutional Review Board approval was waived for the current study as it was an analysis of deidentified secondary data.

### Measures

#### Dependent variable

The dependent variable, loneliness, was measured by the 4-item PROMIS Social Isolation on a 5-point Likert scale response; were: 1 =Never; 2 =Rarely; 3 =Sometimes; 4 =Usually; and 5 =Always. This scale comprises of the following four questions: 1) I feel left out; 2) I feel that people barely know me; 3) I feel isolated from others; and 4) I feel people are around me but not with me. The loneliness scores are calculated only if the respondent answered all four questions. The item scores are summed to create a composite score and then transformed to a t-score (mean=50; standard deviation=10) following recommended scoring instructions [34]. Additional information about this measure can be found in the HINTS 2022 documentation [33]. Higher scores indicate higher levels of loneliness. The 4-item scale had excellent internal consistency as the value for Cronbach’s Alpha for this sample was α= .92.

### Independent variable

The independent variable was the frequency of social media use in the past 12 months. Participants were provided with a list of social media platforms (e.g., Facebook, Twitter, TikTok, YouTube, Instagram) and asked, *“In the last 12 months, how often did you visit a social media site?”* Response options were as follows: Almost every day; At least once a week; A few times a month; Less than once a month; and Never. Responses were collapsed to Almost Daily= 2, Sometimes= 1, and Never= 0 because of low frequency distribution in some responses. More specifically, the response options of “At least once a week”, “A few times a month”, and “Less than once a month” were combined to create the “Sometimes” category due to the proportion of those groups being 12 %, 7.7 %, and 6.2 %, respectively. The collapsing of this variable was used in a similar study in the same manner [21].

### Covariates

In addition to focusing on the relationship between social media use and loneliness, several other variables were considered as covariates. These variables, or a proxy, have been used in other studies focused on social media use and loneliness [21,35,36]. The demographic variables included age, gender, race and ethnicity, education, and income. There were three variables capturing companionship, including living with other adults, marital status, and having a friend to talk to about their health. We also include self-rated health. Lastly, we included two patient reported outcomes (PROs) capturing scores on measures of meaning and purpose in life (PROMIS Meaning and Purpose; [34]) and anxiety and depression (Patient Health Questionnaire 4 [PHQ-4]; [37]).

### Demographics

The sex of participants was assessed by asking respondents to report their sex at birth as either Male or Female.

The age cohorts of interest were Millennials (ages 26–41 years), Generation X (ages 42–57 years), Baby Boomers (ages 58–76 years), and Silent Generation (ages 77–93 years). Respondents were grouped based on cohorts defined by the Pew Research Center [38]. Age was also included as a continuous variable within each stratified analysis. Gen Z (n = 280) was also present in the data; however, they were excluded from the sample given that: 1) a portion of this group was of college age, which conflicts with other key sociodemographic factors included in the model (e.g., education level, income, marital status) and 2) 90 % of Gen Z reports using social media almost daily and only two cases (2.8 %) reported never using social media, which leaves little variation to use.

Racial and ethnic groups in the sample included those who self-identified as Asian, Black, Hispanic, White, and other. The Asian ethnic group included individuals who self-identified as Asian Indians, Chinese, Filipino, Japanese, Korean, Vietnamese, and other Asian. The Hispanic group included those identifying as Mexican, Puerto Rican, Cuban, other Hispanic, and multiple Hispanic ethnicities. The other group consisted of individuals identifying as Native Hawaiian, Guamanian or Chamorro, Samoan, or other Pacific Islander.

Education was originally coded into four categories (i.e., Less than high school, High school graduate, Some college, and College graduate).

The data included a total household income variable with five categories, including < US $20,000, US $20,000-$34,999, US $35,000-$49,999, US $50,000-$74,999, and ≥US $75,000.

The general health question asked, “*In general, would you say your health is…*” and was self-reported as Excellent, Very Good, Good, Fair, and Poor. Responses were recoded to combine Poor/Fair/Good vs. Very good/Excellent self-rated health.

### Companionship

We used three variables to measure different types of companionship. First, we captured if the respondent lived with at least one other adult (18 years or older). This was a dichotomous variable with No as the reference group. The second variable was marital status. There were six categories that were recoded into Married/living together vs. Not. For the Not category, we combined divorced, widowed, separated, and single/never married. The third variable was a question asking, “Do you have friends or family members that you talk to about your health?” The response options were Yes or No (reference group). These three variables were assessed for multicollinearity, and it was determined that they were acceptable to add as unique variables, with VIF values below 10 and tolerance values above 0.1 [39]. The VIF values were 1.86 1.93, and 1.05 and the tolerance values were 0.54, 0.52, and 0.96, respectively.

### Meaning and purpose

The PROMIS Meaning and Purpose t-score was derived from answers to four items. Participants were asked to rate responses to the following: 1) My life has meaning; 2) I have a clear sense of direction in life; 3) I experience deep fulfillment in my life; and 4) My life has purpose. Response options for each item were: 1 =Very much, 2 =Quite a bit, 3 =Somewhat, 4 =A little bit, and 5 =Not at all. The score was only derived if the respondent answered all four questions. The reverse scores of these questions were summed and then transformed to a t-score (mean=50; standard deviation=10) using recommended scoring instructions [34]. Additional information about this measure can also be found in the HINTS 2022 documentation [33]. A higher score indicates feeling a stronger sense of meaning and purpose in life. The 4-item scale had excellent internal consistency, with α= .93 for this sample.

### Depression and anxiety

The PHQ-4 total score was derived from answers to four questions: *Over the past 2 weeks, how often have you been bothered by:* 1) Little interest or pleasure in doing things; 2) Feeling down, depressed, or hopeless; 3) Feeling nervous, anxious or on edge; and 4) Not being able to stop or control worrying. Response options for each item were: 1 =Nearly every day, 2 =More than half the days, 3 =Several days, and 4 =Not at all. The score was derived only if the respondent answered all four questions. The total score was created by 1) rescoring the variables to 0–3 and then reverse coding such that Not at all= 0, Several Days= 1, More than half the days= 2, Nearly every day= 3. Then, 2) computing the total score by summing across the four items. The possible range of total score was from 0 to12 [37]. A higher score indicates feeling more anxious and depressed. The 4-item scale had excellent internal consistency, with α= .86 for this sample.

### Statistical analyses

The analyses were conducted using SAS 9.3 [40]. Complete case analysis was used. This study used PROC SURVEYLOGISTIC with replicate weights and the jackknife approach, as suggested by the HINTS documentation, to provide population-level results adjusted for the sampling methods [33]. Sample descriptive characteristics were examined using frequencies, percentages, means, and standard deviations. This was done with PROC SURVEYFREQ and SURVEYMEANS with the final sample weight. Chi-squared and ANOVA tests were conducted to assess differences in all variables of interest by age cohort.

Variables that had missing data greater than 10 % were income (13 %) and race/ethnicity (12 %). Gender, education, and marital status were missing data for 7 % of respondents and all other variables had only 5 % or less missing data. Given that the key variables of interest (social media use, age, and loneliness) had only 5 % or less missing data, we used the list-wise deletion method to handle missing data. The final analytic sample included 4774 respondents. Additional sensitivity analyses were conducted to assess if missing data was due to a systematic issue, which showed that there was not a significant systematic difference.

This study used multiple linear regression stratified by four age cohorts to examine the association of social media use with loneliness and to assess differences across age cohorts. With the pooled sample, we assessed differences in loneliness across age cohorts and tested an interaction between age cohort and social media use. Unstandardized betas, standard errors, and p-values with alpha < .05 were used to assess effect size and significance.

## Results

### Descriptive characteristics

Table 1 presents descriptive statistics for the final analytic sample and stratification by age cohort. Notably, Chi-squared and ANOVA tests showed significant differences across age cohorts for all variables of interest. The distribution of males and females was about equal except for a slightly lower proportion of men (46 %) in the Silent Generation sample. The proportion of White respondents increased from youngest (52 %) to oldest age (82 %). The proportion of those attaining some college or more decreased with increasing age cohort (79–69 %). The proportion of respondents earning less than $20,000 was relatively stable across age (~12 %). The proportion of those living alone increased with age and ranged from 18 % to 35 %. Very Good to Excellent self-reported health decreased with increasing age cohort, ranging from 52 % to 37 %.

The PROMIS meaning and purpose scores and the PHQ-4 scores were relatively stable with minor significant differences, where the younger age groups had scores representing more meaning and purpose but also more anxiety and depression. Overall, all age groups had PROMIS meaning and purpose scores above the t-score mean, representing having more meaning and purpose (~M=55, SD=10). The range of the t-scores for this sample was from 21.2 to 65.5. For the PHQ-4 scores, Millennials and Generation X had scores approaching the cut-off score (3.0) for a positive screening of anxiety and depression. Almost daily social media use decreased with age (72–26 %) and most respondents within each cohort used social media almost daily or sometimes, except in the Silent Generation where the proportions of never users (48 %) to almost daily and sometimes combined were nearly equal. The mean PROMIS social isolation score decreased with age, ranging from 48.2 (SD=10.1) to 44.4 (SD=8.4), such that older cohorts reported less loneliness. Table 2 provides the mean PROMIS social isolation score for each variable, including PROMIS meaning and purpose and PHQ-4 which were dichotomized based on the cut-off score in the literature.

### Multiple linear regression

#### Pooled sample

Table 3 presents results from multiple linear regression analyses that examined the associations between social media use and loneliness from the pooled sample and sample stratified by age cohort. Compared to never using social media, using social media sometimes (*b*=1.25, *p* = .008) and almost daily (*b*=1.06, *p* = .02) were associated with higher loneliness scores in the pooled sample. Results showed that compared to being a Millennial, being a Baby Boomer (*b*=−1.09, *p* = .02) and from the Silent Generation (*b*=−1.82, *p* = .01) were associated with lower loneliness scores. Being Latino compared to White was associated with having a 1.22-point lower loneliness score (*p* = .02). Compared to attaining less than high school education, attaining some college (*b*=1.76, *p* = .04) and a college degree (*b*=2.33, *p* = .01) were associated with higher loneliness scores, respectively. Compared to reporting an annual household income of less than $20,000, reporting an annual household income of $20,000 to $34,999 was associated with a 1.84-point lower loneliness score (*p* = .004). Compared to not being married or living together, those who are married or living together had a lower loneliness score (*b*=−1.17, *p* = .01). Compared to reporting having no friends or family to talk to about health, reporting having friends or family to talk to about health was associated with a 1.04-point lower loneliness score (*p* = .02). Every one unit increase in PROMIS Meaning and Purpose score was associated with a 0.32-point decrease in loneliness score (*p* < .001). Every one unit increase in PHQ-4 was associated with a 1.37-point increase in loneliness score (*p* < .001). There was no significant interaction between age cohort and social media use (B=1.21; p = 0.32; not shown in Table 2), thus, further post hoc analyses were not pursued.

### Millennials

Social media use was not associated with loneliness score. On average, being Hispanic compared to White was associated with having a 1.94-point lower loneliness score (*p* = .03). Compared to reporting an annual household income of less than $20,000, reporting an annual household income of $20,000 to $34,999 was associated with a 3.32-point lower loneliness score (*p* = .04). Every one unit increase in PROMIS Meaning and Purpose score was associated with a 0.24-point decrease in loneliness score (*p* < .001). Every one unit increase in PHQ-4 was associated with a 1.76-point increase in loneliness score (*p* < .001).

### Generation X

Social media use was not associated with loneliness score. Compared to those reporting not living with any other adults in the household, reporting living with at least one other adult was associated with a lower loneliness score (*b*=−2.33, *p* = .02). Every one unit increase in PROMIS Meaning and Purpose score was associated with a 0.36-point decrease in loneliness score (*p* < .001). Every one unit increase in PHQ-4 was associated with a 1.05-point increase in loneliness score (*p* < .001). Compared to reporting having no friends or family to talk to about health, reporting having friends or family to talk to about health was associated with a 1.83-point lower loneliness score (*p* = .04).

### Baby boomers

Compared to never using social media, using social media almost daily (*b*=1.53, *p* = .008) was associated with a higher loneliness score. Compared to not being married or living together, those who are married or living together had a lower loneliness score (*b*=−1.92, *p* = .008). Every one unit increase in PROMIS Meaning and Purpose score was associated with a 0.32-point decrease in loneliness score (*p* < .001). Every one unit increase in PHQ-4 was associated with a 1.32-point increase in loneliness score (*p* < .001).

### Silent generation

Compared to never using social media, using social media almost daily (*b*=2.43, *p* = .04) was associated with a higher loneliness score. Compared to reporting poor/fair/good health, reporting Very good/Excellent health was associated with a 1.89-point lower loneliness score (*p* = .03). Every one unit increase in PROMIS Meaning and Purpose score was associated with a 0.32-point decrease in loneliness score (*p* < .001). Every one unit increase in PHQ-4 was associated with a 1.19-point increase in loneliness score (*p* < .001). Comparing generations, only baby boomers and the silent generation experienced significant associations with social media use and both in the same direction.

## Discussion

The current study examined the relationship between social media use and loneliness; and whether this relationship differed across age cohorts. There was a significant relationship between social media use and loneliness in the full analytic sample. Descriptively, almost daily social media use decreased with increasing age by cohort. Additionally, the average PROMIS social isolation scores decreased with age, suggesting, on average, older cohorts reported less loneliness. Social media use at any frequency, compared to never, was associated with higher loneliness. While there was a significant finding indicating older cohorts felt less lonely, the interaction between cohort and social media use, was not significant, suggesting that variation in loneliness by age group may not depend on social media use.

Among Baby Boomers and Silent Generation cohorts, more frequent social media use was associated with higher loneliness scores. This finding is somewhat unexpected, as previous studies have found that social media use is linked to lower levels of loneliness and isolation in older adults [13,18,19]. However, some studies found no evidence of social benefits from social media use in this population [41–43]. It is important to note that the social media effects are compared to no use; thus, it is likely that using no use as the reference group may have contributed to some of our non-significant findings for Millennials and Baby Boomers, since the ‘no use’ group sizes were small resulting in higher SEs for these estimates.

These mixed findings may stem in part from limited or missing data on how frequently and in what ways older adults engage with social media. Among adolescents, research has consistently shown that excessive or problematic social media use is associated with higher levels of isolation and loneliness [11,44–47]. The finding for Baby Boomers and Silent Generation in relation to social media use and loneliness should be interpreted with caution, as the frequency data in this study are coarse and do not clearly distinguish between regular and excessive/problematic use, and directionality cannot be established in this study. Future studies should examine these relationships in longitudinal studies. Beyond frequency, the nature of social media engagement may shape its association with isolation and loneliness, regardless of age group [9,12]. Future research should incorporate more nuanced measures of social media use to differentiate passive consumption from active engagement with others via social media.

The current study found identifying as Hispanic was associated with less loneliness compared to non-Hispanic Whites, but only in the full sample and Millennial group. Most studies find racial/ethnic minority groups feel lonelier, compared to Whites, though this is typically found in older adult samples [48–51].

Better self-rated health is typically associated with lower loneliness [52], but this was only found in the Silent Generation. Companionship was significant for Generation X, as living with at least one other adult and having a friend to talk to about health was significant for this group. The literature has found companionship variables to be significant for the oldest old age groups [54]. It is likely that the many limitations of the study design and use of secondary data led to the non-findings here and elsewhere; thus, future studies need to confirm these findings. Marital status was significant in the full sample and for Baby Boomers, which aligned with the literature that married individuals feel less lonely than non-married individuals [53]. Lastly, having a sense of meaning and purpose were significant factors against loneliness and anxiety and depression were strong risk factors for loneliness. These findings were consistent in all models and align with the extant literature [41,54].

This study includes several limitations. Social media use was self-reported, which may have led to over- or under-reporting. The specific type of social media was not identified, which may provide evidence that a specific platform (or combination of many platforms) may be responsible for feeling lonely. This is an opportunity for future work. Lastly, the study included proxy measures for companionship and social structure but limited direct measures to identify the structure or quality aspects of the participants’ social connection. Despite these limitations, the use of nationally representative sample and stratified analyses by age cohorts provide in-depth understanding of the link between social media use and loneliness, and distinct factors related to loneliness across different age groups.

## Conclusions

The current study used three PROs to help identify ways to improve PROs related to mental health and loneliness. As discussed above, clinical interventionists looking to address PROs with technology-based interventions using social media may borrow from these findings and adjacent literature to produce hypotheses that go beyond the limitations of the current study. Specifically, researchers who are looking to address loneliness using social media, can use the findings to further assess social media use as a risk or protective factor for loneliness for those who are from the Baby Boomer and Silent Generation. Studies are needed to confirm the directionality of social media use and loneliness. The results of the present study correspond to the literature indicating that all age groups suffer from loneliness to some extent and further development of social media-based interventions require additional evaluation of their effectiveness across generations. The ubiquity of technology is not decreasing. Finding innovative ways to leverage technology and social media use to improve PROs for mental health is warranted.

## Figures and Tables

**Table 1:** Descriptive characteristics of study sample by age cohort, HINTS 2022, (n = 4774).

	Final Analytic Sample *n*(%) *M*/*SD*	Millennials *n*(%) *M*/*SD*	Generation X *n*(%) *M*/*SD*	Baby Boomers *n*(%) *M*/*SD*	Silent Generation *n(%)* *M/SD*	Chi-squared or ANOVA p-value
Sex						.021
Male	1941 (48.8)	422 (49.8)	474 (48.1)	862 (49.3)	183 (45.8)	
Female	2833 (51.2)	660 (50.2)	776 (51.9)	1151 (50.7)	246 (54.2)	
Age	56.2 (15.8)	34.0 (4.4)	50.1 (4.7)	66.5 (5.2)	81.8 (4.1)	< .001
Race/ethnicity						< .001
White	2796 (63.4)	520 (51.5)	679 (64.1)	1281 (70.7)	316 (82.4)	
Black	751 (11.2)	154 (10.9)	207 (12.1)	346 (11.7)	44 (4.7)	
Hispanic	812 (15.0)	263 (20.6)	247 (14.8)	254 (10.7)	48 (8.6)	
Asian	250 (5.5)	97 (8.9)	75 (5.4)	67 (2.9)	11 (1.3)	
Other	165 (5.0)	48 (8.1)	42 (3.6)	65 (4.1)	10 (2.9)	
Education						< .001
Less than high school	269 (5.9)	42 (3.6)	75 (7.5)	116 (6.0)	36 (7.2)	
High school graduate	807 (20.4)	132 (17.4)	203 (20.0)	370 (23.7)	102 (22.0)	
Some college	1352 (38.0)	254 (32.0)	363 (40.2)	608 (39.6)	127 (46.2)	
College graduate	2346 (35.7)	654 (47.0)	609 (32.2)	919 (30.8)	164 (24.6)	
Household Income						< .001
< $20,000	738 (12.4)	135 (12.1)	179 (12.1)	333 (12.8)	91 (14.2)	
$20,000–$34,999	612 (10.6)	100 (9.2)	138 (9.6)	293 (12.1)	81 (16.0)	
$35,000–$49,999	617 (11.9)	125 (13.1)	125 (8.1)	301 (14.6)	66 (14.8)	
$50,000–$74,999	830 (18.1)	201 (18.1)	208 (16.9)	351 (19.0)	70 (21.2)	
$75,000 +	1977 (47.0)	521 (47.5)	600 (53.3)	735 (41.4)	121 (33.9)	
Self-reported health						< .001
Poor/Fair/Good	2617 (53.2)	534 (48.0)	704 (54.4)	1100 (54.9)	279 (63.1)	
Very Good/Excellent	2157 (46.8)	548 (52.0)	546 (45.6)	913 (45.1)	150 (36.9)	
Number of Adults in HH						< .001
Living alone	1585 (20.7)	250 (17.8)	320 (16.0)	797 (26.3)	215 (34.7)	
At least two adults in HH	3192 (79.3)	832 (82.2)	930 (84.0)	1216 (73.7)	214 (65.3)	
Marital Status						< .001
Single/live alone	2167 (37.5)	423 (45.7)	486 (30.0)	1003 (36.2)	255 (47.0)	
Married/living together	2607 (62.5)	659 (54.3)	764 (70.0)	1010 (63.8)	174 (53.0)	
Has friend to talk to about health						< .001
No	851 (19.5)	232 (23.7)	240 (19.3)	333 (17.4)	46 (10.7)	
Yes	3923 (80.5)	850 (76.3)	1010 (80.7)	1680 (82.6)	383 (89.3)	
PROMIS Meaning and purpose	55.0 (10.1)	53.9 (10.4)	54.6 (10.1)	55.8 (9.8)	55.4 (9.8)	< .001
PHQ−4	2.2 (2.8)	2.9 (3.0)	2.4 (3.0)	1.7 (2.5)	1.8 (2.5)	< .001
Social Media Use						< .001
Never	895 (16.1)	50 (7.5)	114 (9.5)	535 (26.3)	196 (47.6)	
Sometimes	1266 (26.4)	194 (20.5)	339 (29.0)	614 (29.4)	119 (26.1)	
Almost daily	2613 (57.5)	838 (72.1)	797 (61.4)	864 (44.2)	114 (26.3)	
PROMIS Social Isolation	46.0 (9.5)	48.2 (10.1)	46.6 (9.6)	44.8 (8.9)	44.4 (8.4)	< .001

HH=household. PROMIS=Patient-Reported Outcomes Measurement Information System, PHQ-4 =Personal Health Questionnaire 4.

**Table 2:** Mean PROMIS social isolation score by each included variable (except age).

	PROMIS Social Isolation Score *M* (*SD*)	p-value
Sex		< .0001
Male	45.2 (9.4)	
Female	46.5 (9.6)	
Race/ethnicity		.0012
White	46.4 (9.0)	
Black	45.5 (10.2)	
Hispanic	45.2 (10.2)	
Asian	45.8 (8.8)	
Other	47.8 (10.3)	
Education		.309
Less than high school	45.5 (10.6)	
High school graduate	45.6 (10.0)	
Some college	46.1 (9.9)	
College graduate	46.1 (8.9)	
Household Income		< .0001
< $20,000	48.2 (11.3)	
$20,000–$34,999	46.7 (9.9)	
$35,000–$49,999	46.4 (9.3)	
$50,000–$74,999	46.2 (9.2)	
$75,000 +	45.0 (8.7)	
Self-reported health		< .0001
Poor/Fair/Good	47.5 (9.9)	
Very Good/Excellent	44.1 (8.7)	
Number of Adults in HH		< .0001
Living alone	47.2 (9.8)	
At least two adults in HH Marital Status	45.3 (9.3)	
Single/live alone	47.5 (9.9)	< .0001
Married/living together	44.6 (8.9)	
Has friend to talk to about health		.0034
No	46.8 (10.9)	
Yes	45.8 (9.2)	
PROMIS Meaning and purpose score		< .0001
≤50	53.1 (8.9)	
51 +	43.2 (8.3)	
PHQ−4 score		< .0001
≤6	44.7 (8.8)	
7 +	57.5 (8.8)	
Social Media Use		< .0001
Never	44.1 (9.4)	
Sometimes	45.9 (9.3)	
Almost daily	46.7 (9.6)	

HH=household. PROMIS=Patient-Reported Outcomes Measurement Information System, PHQ-4 =Personal Health Questionnaire 4.

**Table 3:** Multiple linear regression for PROMIS social isolation stratified by cohort, n = 4774.

	Pooled Sample	Millennials	Generation X		Baby Boomers	Silent Generation
Variable	B (SE)	B (SE)	B (SE)		B (SE)	β SE
Intercept	61.98*** (1.57)	64.72*** (4.12)	67.09*** (4.23)		59.02*** (4.21)	59.94*** (10.37)
Sex						
Male	-	-	-		-	-
Female	0.24 (0.30)	0.05 (0.61)	0.30 (0.68)		0.35 (0.41)	−0.54 (0.98)
Age (continuous)		−0.09 (0.09)	−0.02 (0.06)		−0.01 (0.05)	0.00 (0.11)
Millennials	-	-	-		-	-
Generation X	−0.80 (0.48)					
Baby Boomers	−1.09* (0.43)					
Silent Generation	−1.82** (0.67)					
Race/Ethnicity						
White	-	-	-		-	-
Black	0.16 (0.65)	−0.35 (1.11)	0.39 (1.20)		0.70 (1.02)	0.57 (1.28)
Latino	−1.22* (0.51)	−1.95* (0.88)	−0.96 (0.99)		−0.78 (0.67)	1.75 (1.37)
Asian	−0.90 (0.56)	−0.72 (0.81)	−1.17 (1.01)		−0.82 (1.39)	2.25 (3.23)
Other	0.75 (1.00)	1.22 (1.53)	0.18 (1.55)		0.47 (1.30)	−2.48 (2.16)
Education						
Less than high school	-	-	-		-	-
High school	1.41 (0.88)	−3.46 (2.51)	3.37** (0.96)		1.64 (1.25)	−0.23 (1.98)
Some college	1.76* (0.82)	−2.87 (2.42)	3.63** (1.06)		2.00 (1.29)	−0.60 (1.84)
College graduate	2.33* (0.89)	−3.94 (2.39)	4.97*** (1.19)		2.83* (1.26)	1.13 (1.95)
Household income						
< $20,000	-	-	-	-	-	-
$20,000-$34,999	−1.84** (0.60)	−3.32* (1.56)	−3.39*	1.62	−0.17 (0.92)	1.70 (1.49)
$35,000-$49,999	−1.47 (0.73)	−1.59 (1.23)	−4.01*	1.56	0.10 (1.24)	2.77 (1.67)
$50,000-$74,999	−1.00 (0.66)	0.21 (1.14)	−3.14	1.57	−0.51 (1.18)	1.89 (1.52)
$75,000 +	−1.12 (0.74)	0.01 (1.23)	−3.53*	1.66	−0.13 (1.15)	1.99 (1.42)
Self-rated health						
Poor/fair/good	-	-	-		-	-
Excellent/very good	−0.83 (0.42)	−0.49 (0.72)	−1.24 (0.76)		−0.50 (0.52)	−1.89* (0.85)
Number of Adults in HH						
Living alone	-	-	-		-	-
At least two adults	−0.55 (0.47)	−0.25 (0.90)	−2.33* (0.95)		0.23 (0.70)	−0.74 (1.14)
Marital status						
Single	-	-	-		-	-
Married/living together	−1.17* (0.46)	−0.92 (0.80)	0.30 (0.81)		−1.92** (0.70)	−2.30 (1.34)
Has friend to talk to about health						
No	-	-	-		-	-
Yes	−1.04* (0.44)	−0.95 (0.84)	−1.83* (0.85)		−0.18 (0.74)	−0.36 (1.79)
PROMIS Meaning score	−0.32*** (0.02)	−0.24*** (0.04)	−0.36*** (0.04)		−0.32*** (0.03)	−0.32*** (0.05)
PHQ−4 score	1.37*** (0.09)	1.76*** (0.16)	1.05*** (0.13)		1.32*** (0.14)	1.19*** (0.21)
Social media use						
Never	-	-	-		-	-
Sometimes	1.25* (0.45)	0.55 (1.14)	1.30 (1.16)		0.99 (0.66)	1.14 (1.06)
Almost daily	1.06* (0.42)	0.18 (1.05)	0.45 (1.03)		1.53** (0.56)	2.43* (1.14)

P value:

* < 0.05,

** < 0.01,

*** < 0.001;

B=Beta, SE=Standard Error, PROMIS=Patient-Reported Outcomes Measurement Information System, PHQ-4 =Personal Health Questionnaire 4.

## Data Availability

The data that support the findings of this study are openly available at https://hints.cancer.gov/data/download-data.aspx.

## Impact statement

This study advances the science and application of PROMs by using the PROMIS social isolation measure as an outcome with a nationally representative sample and multiple age groups. This measure has not been used in another study with social media as the independent variable.

## References

[1] Czaja SJ, Moxley JH, Rogers WA, Social support, isolation, loneliness, and health among older adults in the PRISM randomized controlled trial, Front. Psychol 12 (2021) 728658, 10.3389/fpsyg.2021.728658. 34675843 PMC8525598

[2] Machell T, Paul E, Zhao G, Thomas CW, Hsia J, Pierannunzi C, Hacker K, Racial and ethnic differences in social determinants of health and health-related social needs among adults - behavioral risk factor surveillance system, United States, MMWR Morb. Mortal. Wkly. Rep (2022), 10.15585/mmwr.mm7309a3. PMC10932584 38451870

[3] Hawkley LC, Buecker S, Kaiser T, Luhmann M, Loneliness from young adulthood to old age: explaining age differences in loneliness, Int. J. Behav. Dev 46 (1) (2022) 39–49, 10.1177/0165025420971048. 35001993 PMC8734589

[4] Nguyen TT, Lee EE, Daly RE, Wu TC, Tang Y, Tu X, Van Patten R, Jeste DV, Palmer BW, Predictors of loneliness by age decade: study of psychological and environmental factors in 2,843 community-dwelling Americans aged 20–69 years, J. Clin. Psychiatry 81 (6) (2020) 15111, 10.4088/JCP.20m13378. PMC7953851 33176072

[5] Bruss KV, Seth P, Zhao G, Loneliness, lack of social and emotional support, and mental health issues — United States, 2022, MMWR Morb. Mortal. Wkly. Rep 73 (2024) 539–545, 10.15585/mmwr.mm7324a1. 38900690 PMC11199020

[6] Barreto M, Victor C, Hammond C, Eccles A, Richins MT, Qualter P, Loneliness around the world: age, gender, and cultural differences in loneliness, Pers. Individ. Differ 169 (2021) 110066, 10.1016/j.paid.2020.110066. PMC7768187 33536694

[7] WHO. Commission on Social Connection. 2023. <https://www.who.int/groups/commission-on-social-connection> (accessed 13 November 2024).

[8] Mohd Tohit NF, Haque M, Preparing the younger generation for an aging society: strategies, challenges, and opportunities, Cureus 16 (7) (2024) e64121, 10.7759/cureus.64121. 38983672 PMC11231670

[9] Hutto CJ, Bell C, Farmer S, Fausset C, Harley L, Nguyen J, Fain B, Social media gerontology: understanding social media usage among older adults, Web Intell. 13 (2015) 69–87, 10.3233/WEB-150310.

[10] National Academies of Sciences, Engineering, and Medicine, Social Isolation and Loneliness in Older Adults: Opportunities for the Health Care System, The National Academies Press, Washington, DC, 2020, 10.17226/25663. 32510896

[11] General U.S. Our epidemic of loneliness and isolation. The US Surgeon General’s Advisory on the Healing Effects of Social Connection and Community 2023. <https://www.hhs.gov/sites/default/files/surgeon-general-social-connection-advisory.pdf> (accessed 13 November 2024).

[12] Verduyn P, Ybarra O, Résibois M, Jonides J, Kross E, Do social network sites enhance or undermine subjective well-being? a critical review, Soc. Issues Policy Rev 11 (1) (2017) 274–302, 10.1111/sipr.12033.

[13] Zhang K, Kim K, Silverstein NM, Song Q, Burr JA, Social media communication and loneliness among older adults: the mediating roles of social support and social contact, Gerontologist 61 (6) (2021) 888–896, 10.1093/geront/gnaa197. 33284972

[14] Panayiotou M, Badcock JC, Lim MH, Banissy MJ, Qualter P, Measuring loneliness in different age groups: the measurement invariance of the UCLA loneliness scale, Assessment 30 (5) (2023) 1688–1715, 10.1177/10731911221119533. 36031881 PMC10248311

[15] Wu P, Feng R, Zhang J, The relationship between loneliness and problematic social media usage in Chinese university students: a longitudinal study, BMC Psychol. 12 (1) (2024) 13, 10.1186/s40359-023-01498-4. 38178215 PMC10765645

[16] Francis J, Brauer S, Social isolation, loneliness, and mattering among older adult facebook users from diverse backgrounds, 33–33, Innov. Aging 6 (Supplement_1) (2022), 10.1093/geroni/igac059.122.

[17] McWhorter RR, Delello JA, Gipson CS, Mastel-Smith B, Caruso K, Do loneliness and social connectedness improve in older adults through mobile technology? in: Delello JA, McWhorter RR (Eds.), Disruptive and Emerging Technology Trends Across Education and the Workplace, IGI Global, 2020, pp. 221–242, 10.4018/978-1-7998-2914-0.ch009.

[18] Kusumota L, Diniz MAA, Ribeiro RM, Silva ILCD, Figueira ALG, Rodrigues FR, Rodrigues RAP, Impact of digital social media on the perception of loneliness and social isolation in older adults, Rev. Lat. Am. Enferm 30 (2022) e3573, 10.1590/1518-8345.5641.3573. PMC9132132 35613252

[19] Chopik WJ, The benefits of social technology use among older adults are mediated by reduced loneliness, Cyber Behav. Soc. Netw 19 (9) (2016) 551–556, 10.1089/cyber.2016.0151. PMC5312603 27541746

[20] Wang YL, Chen YJ, Liu CC, The relationship between social media usage and loneliness among younger and older adults: the moderating effect of shyness, BMC Psychol. 12 (1) (2024) 343, 10.1186/s40359-024-01727-4. 38863021 PMC11167928

[21] Primack BA, Shensa A, Sidani JE, Whaite EO, yi Lin L, Rosen D, Colditz JB, Radovic A, Miller E, Social media use and perceived social isolation among young adults in the US, Am. J. Prev. Med 53 (1) (2017) 1–8, 10.1016/j.amepre.2017.01.010. 28279545 PMC5722463

[22] Hunt MG, Marx R, Lipson C, Young J, No more FOMO: limiting social media decreases loneliness and depression, J. Soc. Clin. Psychol 37 (10) (2018) 751–768, 10.1521/jscp.2018.37.10.751.

[23] Azhari A, Toms Z, Pavlopoulou G, Esposito G, Dimitriou D, Social media use in female adolescents: associations with anxiety, loneliness, and sleep disturbances, Acta Psychol. 229 (2022) 103706, 10.1016/j.actpsy.2022.103706. 35973307

[24] Casanova G, Zaccaria D, Rolandi E, Guaita A, The effect of information and communication technology and social networking site use on older people’s well-being in relation to loneliness: review of experimental studies, J. Med. Internet Res 23 (3) (2021) e23588, 10.2196/23588. 33439127 PMC7961406

[25] Lewin KM, Meshi D, Schuster AM, Cotten SR, Active and passive social media use are differentially related to depressive symptoms in older adults, Aging Ment. Health 27 (1) (2023) 176–183, 10.1080/13607863.2022.2068133. 35470731

[26] Batra R, Flatt JD, Pharr JR, Sharma M, Khubchandani J, Kanekar A, Chirico F, Batra K, Exploring social support strategies and socio-cultural factors influencing social isolation and loneliness: the role of digital literacy, Healthcare (Basel) 12 (21) (2024) 2149, 10.3390/healthcare12212149. 39517361 PMC11544966

[27] Shah HA, Househ M, Understanding loneliness in younger people: review of the opportunities and challenges for loneliness interventions, Interact. J. Med. Res 12 (2023) e45197, 10.2196/45197. 37917125 PMC10654910

[28] Stanton AG, Jerald MC, Ward LM, Avery LR, Social media contributions to strong black woman ideal endorsement and black women’s mental health, Psychol. Women Q 41 (4) (2017) 465–478, 10.1177/0361684317732330.

[29] Woods HC, Scott H, #Sleepyteens: social media use in adolescence is associated with poor sleep quality, anxiety, depression and low self-esteem, J. Adolesc 51 (1) (2016) 41–49, 10.1016/j.adolescence.2016.05.008. 27294324

[30] Auxier B, Anderson M, Social media use in 2021, Pew Res. Cent 1 (1) (2021) 1–4 <https://www.pewresearch.org/internet/2021/04/07/social-media-use-in-2021/> (accessed 13 November 2024)..

[31] Faverio M, Share of those 65 and older who are tech users has grown in the past decade, Pew Res. Cent 13 (7) (2022), <https://www.pewresearch.org/short-reads/2022/01/13/share-of-those-65-and-older-who-are-tech-users-has-grown-in-the-past-decade/> (accessed 13 November 2024)..

[32] Gottfried J, Americans’ social media use, Pew Res. Cent 31 (2024), <https://www.pewresearch.org/internet/2024/01/31/americans-social-media-use/> (accessed 13 November 2024)..

[33] Westat. HINTS 6 Methodology Report. Health Information National Trends Survey 6 (HINTS 6). National Cancer Institute. 2023. <https://hints.cancer.gov/data/download-data.aspx#H6>.

[34] Hahn EA, DeVellis RF, Bode RK, Garcia SF, Castel LD, Eisen SV, Bosworth HB, Heinemann AW, Rothrock N, Cella D, PROMIS cooperative group. Measuring social health in the patient-reported outcomes measurement information system (PROMIS): item bank development and testing, Qual. Life Res 19 (2010) 1035–1044, 10.1007/s11136-010-9654-0. 20419503 PMC3138729

[35] Sewall CJ, Goldstein TR, Wright AG, Rosen D, Does objectively measured social-media or smartphone use predict depression, anxiety, or social isolation among young adults? Clin. Psychol. Sci 10 (5) (2022) 997–1014, 10.1177/21677026221078309. 36406004 PMC9671480

[36] Hajek A, König HH, Online social media use, loneliness and perceived social isolation in later life, in: Hajek A, Riedel-Heller SG, Konig HH (Eds.), Loneliness and Social Isolation in Old Age, 95 Routledge, 2023, < 10.4324/9781003289012>.

[37] Dimock M, Defining generations: where millennials end and generation z begins, Pew Res. Cent (2019), <https://www.pewresearch.org/fact-tank/2019/01/17/where-millennials-end-and-generation-z-begins/> (accessed 13 November 2024)..

[38] Belsley DA, Kuh E, Welsch RE, Regression Diagnostics: Identifying Influential Data and Sources of Collinearity, Wiley, New York, 1980, 10.1002/0471725153.

[39] Kroenke K, Spitzer RL, Williams JB, Löwe B, An ultra-brief screening scale for anxiety and depression: the PHQ–4, Psychosomatics 50 (6) (2009) 613–621, 10.1176/appi.psy.50.6.613. 19996233

[40] SAS Institute Inc. SAS 9.1.3 Help and Documentation. Cary, NC: SAS Institute Inc.; 2002–2004.

[41] Shovestul B, Han J, Germine L, Dodell-Feder D, Risk factors for loneliness: the high relative importance of age versus other factors, PLoS One 15 (2) (2020) e0229087, 10.1371/journal.pone.0229087. 32045467 PMC7012443

[42] Aarts S, Peek STM, Wouters EJM, The relation between social network site usage and loneliness and mental health in community-dwelling older adults, Int. J. Geriatr. Psychiatry 30 (9) (2015) 942–949, 10.1002/gps.4241. 25503540

[43] Wiwatkunupakarn N, Pateekhum C, Aramrat C, Jirapornchaoren W, Pinyopornpanish K, Angkurawaranon C, Social networking site usage: a systematic review of its relationship with social isolation, loneliness, and depression among older adults, Aging Ment. Health 26 (7) (2022) 1318–1326, 10.1080/13607863.2021.1966745. 34427132

[44] Moretta T, Buodo G, Problematic Internet use and loneliness: how complex is the relationship? A short literature review, Curr. Addict. Rep 7 (2020) 125–136, 10.1007/s40429-020-00305-z.

[45] Meshi D, Cotten SR, Bender AR, Problematic social media use and perceived social isolation in older adults: a cross-sectional study, Gerontology 66 (2) (2020) 160–168, 10.1159/000502577. 31522178

[46] Kim YK, Fingerman KL, Daily social media use, social ties, and emotional well-being in later life, J. Soc. Pers. Relatsh 39 (6) (2022) 1794–1813, 10.1177/02654075211067254. PMC10508904 37727534

[47] Macdonald B, Luo M, Hülür G, Daily social interactions and well-being in older adults: the role of interaction modality, J. Soc. Pers. Relatsh 38 (12) (2021) 3566–3589, 10.1177/02654075211052536.

[48] Raymo JM, Wang J, Loneliness at older ages in the United States: lonely life expectancy and the role of loneliness in health disparities, Demography 59 (3) (2022) 921–947, 10.1215/00703370-9937606. 35502830

[49] Taylor HO, Chen YC, Tsuchiya K, Cudjoe TK, Qin W, Nguyen AW, Roy A, Racial/ethnic differences in loneliness among older adults: the role of income and education as mediators, Innov. Aging 8 (8) (2024) igae068, 10.1093/geroni/igae068. 39139381 PMC11319872

[50] Gallegos ML, Segrin C, Family connections and the latino health paradox: exploring the mediating role of loneliness in the relationships between the latina/o cultural value of familism and health, Emergent Health Communication Scholarship from and about African American, Latino/a/x, and American Indian/Alaskan Native Peoples, Routledge, 2024, pp. 148–158, 10.1080/10410236.2021.1909244. 33853460

[51] Tibirica L, Jester DJ, Jeste DV, A systematic review of loneliness and social isolation among Hispanic/Latinx older adults in the United States, Psychiatry Res. 313 (2022) 114568, 10.1016/j.psychres.2022.114568. 35643058

[52] Sol K, Brauer S, Antonucci TC, Longitudinal associations between loneliness and self-rated health among black and White older adults, J. Gerontol B 78 (4) (2023) 639–648, 10.1093/geronb/gbac200. PMC10066738 36571393

[53] Surkalim DL, Clare PJ, Eres R, Gebel K, Bauman A, Ding D, Have middle-aged and older Americans become lonelier? 20-Year trends from the health and retirement study, J. Gerontol. B Psychol. Sci. Soc. Sci 78 (7) (2023) 1215–1223, 10.1093/geronb/gbad062. 37279542 PMC10292834

[54] Kislev E, Aging, marital status, and loneliness: multilevel analyses of 30 countries, Res. Ageing Soc. Policy 10 (1) (2022) 77–103, 10.17583/rasp.8923.

